# Association between apoptosis and mortality in severe septic patients

**DOI:** 10.1186/cc14133

**Published:** 2015-03-16

**Authors:** L Lorente, M Martín, A González-Rivero, J Ferreres, J Solé-Violán, L Labarta, C Díaz, A Jiménez, J Borreguero-León

**Affiliations:** 1 Hospital Universitario de Canarias, La Laguna, Tenerife, Spain; 2 Hospital Universitario Nuestra Señora Candelaria, Santa Cruz, Tenerife, Spain; 3 Hospital Clínico Universitario de Valencia, Spain; 4 Hospital Universitario Dr. Negrín, Las Palmas de Gran Canaria, Spain; 5 Hospital San Jorge, Huesca, Spain; 6 Hospital Insular, Las Palmas de Gran Canaria, Spain

## Introduction

The apoptotic process, in which cells are actively eliminated by a programmed pathway, is increased in sepsis. Extrinsic and intrinsic apoptotic death cell pathways activate caspase-3, which leads to cell apoptosis. Cytokeratin 18 (CK-18), a protein present in most epithelial and parenchymal cells, is cleaved by the action of caspases and released into the blood as caspase-cleaved CK (CCCK)-18 during apoptotic death. The novel objectives of this study were to determine whether there are associations between serum caspase-3 levels, serum CK-18 levels and mortality in septic patients.

## Methods

A prospective, multicenter, observational study in six Spanish ICUs, including 216 patients with severe sepsis. We collected blood samples at the severe sepsis diagnosis moment to determine serum levels of caspase-3 (to assess the main executor of apoptosis) and CCCK18 (to assess the apoptosis level). The endpoint was 30-day mortality.

## Results

We found that nonsurvivor (*n *= 76) in comparison with survivor (*n *= 140) septic patients showed higher serum levels of caspase-3 (0.41 ng/ml (0.14 to 0.52) vs. 0.11 ng/ml (0.10 to 0.25); *P *< 0.001) and CCCK18 (448 (310 to 723) vs. 319 (236 to 445); *P *< 0.001). Multiple logistic regression showed that serum caspase-3 levels >0.25 ng/ml were associated with mortality at 30 days (odds ratio = 6.51; 95% confidence interval = 3.32 to 12.77; *P *< 0.001), controlling for SOFA score and age. Kaplan-Meier survival analysis showed a higher risk of death in septic patients with serum caspase-3 levels >0.25 ng/ml than in patients with lower levels (hazard ratio = 3.80; 95% CI = 2.35 to 6.15; *P *< 0.001). We found a positive association between serum levels of caspase-3 and CCCK-18 (ρ = 0.32; *P *< 0.001).

## Conclusion

The novel findings of our study were that there is an association between serum caspase-3 levels, serum CK-18 levels and mortality in septic patients. There has been reported decreased apoptosis and increased survival in septic rats with the administration of caspase inhibitors; thus, it may be interesting to explore those agents in septic patients.

## Acknowledgements

Funded by Grant FIS-PI-14-00220 from Instituto de Salud Carlos III (Madrid, Spain) and co-financed by Fondo Europeo de Desarrollo Regional (FEDER).

